# Radiation, Immune Checkpoint Blockade and the Abscopal Effect: A Critical Review on Timing, Dose and Fractionation

**DOI:** 10.3389/fonc.2018.00612

**Published:** 2018-12-13

**Authors:** Zachary S. Buchwald, Jacob Wynne, Tahseen H. Nasti, Simeng Zhu, Waleed F. Mourad, Weisi Yan, Seema Gupta, Samir N. Khleif, Mohammad K. Khan

**Affiliations:** ^1^Department of Radiation Oncology, Emory University, Atlanta, GA, United States; ^2^Department of Microbiology and Immunology, Emory University, Atlanta, GA, United States; ^3^Department of Radiation Oncology, Henry Ford Health System, Detroit, MI, United States; ^4^Erlanger UT Radiation Oncology, Chattanooga, TN, United States; ^5^Mitchell Cancer Institute, University of Southern Alabama, Mobile, AL, United States; ^6^Georgia Cancer Center, Augusta University, Augusta, GA, United States

**Keywords:** radiation, immunotherapy, checkpoint blockade, abscopal effect, PD-1, PD-L1

## Abstract

The combination of radiation and immunotherapy is currently an exciting avenue of pre-clinical and clinical investigation. The synergy between these two treatment modalities has the potential to expand the role of radiation from a purely local therapy, to a role in advanced and metastatic disease. Tumor regression outside of the irradiated field, known as the abscopal effect, is a recognized phenomenon mediated by lymphocytes and enhanced by checkpoint blockade. In this review, we summarize the known mechanistic data behind the immunostimulatory effects of radiation and how this is enhanced by immunotherapy. We also provide pre-clinical data supporting specific radiation timing and optimal dose/fractionation for induction of a robust anti-tumor immune response with or without checkpoint blockade. Importantly, these data are placed in a larger context of understanding T-cell exhaustion and the impact of immunotherapy on this phenotype. We also include relevant pre-clinical studies done in non-tumor systems. We discuss the published clinical trials and briefly summarize salient case reports evaluating the abscopal effect. Much of the data discussed here remains at the preliminary stage, and a number of interesting avenues of research remain under investigation.

## Introduction

Traditionally, radiation therapy (RT) is considered a local form of cancer treatment with an “in-field” anti-tumor effect. RT has been used to treat localized malignancies with curative intent or to palliate painful, bleeding or otherwise problematic metastases. Over time radiation delivery has changed (2-D vs. 3-D vs. IMRT), however, the basic philosophy focused on controlling local disease has persisted. Interestingly, in patients with multiple lesions, tumor regression occurs, although rarely, outside the RT field. This is known as an abscopal effect or “ab”- away from, “scopus”–target. This was first described by Mole et al. (1) with over 46 cases of a RT-induced abscopal effect subsequently documented including a prominent report from Memorial Sloan Kettering (2, 3). Patients with several distinct cancer histologies and across a range of ages have benefited from this phenomenon. The abscopal response is now being interrogated with increasing vigor with the goal of improved therapeutic outcomes for metastatic cancer patients, especially in combination with emerging immunotherapy agents (3).

T-cell checkpoints (CTLA-4, PD-1) are cell surface molecules which prevent T-cell activation or reinvigoration following chronic antigen exposure (4–6). Inhibiting these T-cell checkpoints leads to greater anti-tumor T-cell activity. Checkpoint inhibitors are now the most frequently prescribed immunotherapy and have shown great promise in many different malignancies (7–11). Interestingly, the relatively rare abscopal effect has been observed with increasing frequency as checkpoint inhibitors are being given in close temporal proximity or concurrently with RT (12). There are many questions that remain unanswered regarding the safety, efficacy, optimal dose/fractionation and timing of immune-checkpoint inhibitors in combination with RT. Here we present several mechanisms responsible for the abscopal effect and summarize relevant basic science findings, clinical trials, and clinical case reports. We also provide data which may inform optimization of RT dose, fractionation and timing of administration of immune-checkpoint blockade/immuno-modulators in order to maximize the RT-induced abscopal effect.

## Radiation and the Immune System

Classically, RT was thought to be immunosuppressive due to the exquisite radio-sensitivity of leukocytes; but, more recently, data has shown that RT can enhance various components of the antigen processing and presentation pathway (13–15). Reits et al. demonstrated *in vitro* and *in vivo*, a dose dependent increase in cell-surface MHC-I levels in response to RT in a transcription independent manner (16). This is thought to be due to an increased intracellular peptide pool from both increased protein translation and increased protein degradation leading to a larger epitope repertoire to be presented following tumor cell death.

Liberation of antigens and increased MHC-I expression alone, however, would not be sufficient for effective anti-tumor T-cell priming. For this, maturation of antigen presenting cells (APCs) is necessary. APC maturation involves, in addition to MHC-I and II upregulation, increased expression of costimulatory ligands B7-1, B7-2 as well as cytokine production important for T-cell proliferation and phenotypic skewing (17). This can occur via APC pathogen recognition receptor (PRR) ligation by non-self-derived adjuvants, pathogen-associated molecular patterns (PAMPs), or endogenous damage associated molecular patterns (DAMPs) (18). Importantly, RT can induce immunogenic cell death (ICD), which, in contrast to apoptosis, releases tumor cell contents, including DAMPs, in a disorganized fashion which can be highly pro-inflammatory. In the context of RT induced ICD, DAMPs include high-motility group box 1 (HMGB1), heat shock protein 70 (HSP 70), GP96 and calreticulin membrane exposure (19–21). Calreticulin, an endoplasmic reticulum resident molecular chaperone, can stimulate phagocytosis of cancer cells by dendritic cells (22) while HMBG1, a critical chromatin protein, promotes antigen presentation (23). Radiation-induced calrecticulin exposure increases T-cell mediated tumor lysis, and in the presence of a calreticulin-blocking peptide this effect was abrogated (24). Wang et al. have shown that RT, over a wide dose range, induced HMGB1 extracellular release and cytoplasmic translocation in a dose and time-dependent manner (25). The subsequent HMGB1 mediated APC maturation is TLR-4 dependent (26). An integral role for APCs in anti-tumor T-cell priming and the abscopal effect was shown in a bilateral syngeneic mouse model of breast cancer wherein immunoadjuvant treatment with FMS-like tyrosine kinase receptor 3 ligand (FLT3L), which promote DC development and bone marrow egress (27), resulted in growth delay in an irradiated flank tumor as well as the untreated, contralateral tumor (28). Together these data support an intimate relationship between an anti-tumor immune response and RT mediated tumor cell killing.

## Radiation Sequencing With Immunotherapy

How does this immunogenic antigen bolus released by RT and presented by APCs synergize with checkpoint inhibitors to enhance the anti-tumor immune response, and how does this inform the sequencing of these two treatment modalities? Two candidate mechanisms to explain this synergy are proposed: (1) neo-antigens released in response to RT may act in concert with anti-PD-1 immunotherapy to only reinvigorate exhausted intratumoral CD8 T-cells, or (2) RT may stimulate proliferation and differentiation of naïve T-cells in response to liberated neo-antigens while anti-PD-1 may potentiate naïve T-cell activation in addition to reinvigorating exhausted T-cells. Each mechanism leads to a more robust immune response, but would result in a different response amplitude and carries different implications for combined modality therapy. If the immunogenic effect arises from naïve T-cell proliferation and activation, very close sequencing of RT and anti-PD-1 will be required for anti-PD-1 to potentiate early T-cell activation. Whereas, if the reinvigoration of exhausted T-cells is the dominant mechanism, this temporal overlap may be less critical and the effect would be additive rather than synergistic. Current evidence suggests that RT acts primarily to stimulate proliferation and differentiation of naïve T based on a broadening of the T-cell receptor repertoire post-RT although this may reflect an expansion of low frequency exhausted clones (29). These two mechanisms described are not mutually exclusive, however, pre-clinical tumor data has demonstrated that initiating anti-PD-L1 7 days following RT was inferior to starting on either the first or the last day (30). These data support (2), however, a deeper understanding of the underlying mechanism can be found in models of acute viral infections.

The Armstrong strain of lymphocytic choriomeningitis virus (LCMV) is a well characterized system for studying acute T-cell responses and naïve T-cell differentiation (31). In a recently published study, it was demonstrated that exposure to anti-PD-L1 during early T-cell differentiation to an acute Armstrong infection impacts T-cell effector function (32). The authors showed that the acute T-cell response is inhibited by endogenous PD-1 activity, but that anti-PD-L1 during initial T-cell activation increases granzyme B expression in virus-specific CD8 T-cells, resulting in faster clearance of infection (32). While the role of PD-1 in mediating CD8 T-cell reinvigoration in chronic infection is well established (33), this finding supports previous reports by Barber et al. that showed in acute infection of PD-L1^−/−^ mice with LCMV resulted in a heightened CD8 T-cell response. Furthermore, both found the CD8 T-cell response was also improved in chronic infection characterized by T-cell exhaustion (6). Together, these data support close sequencing of RT and checkpoint blockade as late administration of anti-PD-L1 reinvigorates exhausted T-cells without the added benefit of influencing initial T-cell activation and differentiation. Data to directly support these findings in a tumor model combining RT and checkpoint blockade is still lacking.

The kinetics of T-cell tumor infiltration following RT also helps inform the sequencing and timing of anti-PD-1/L1 administration. Following tumor irradiation with 12 Gy on 2 consecutive days, it was shown that overall leukocyte and CD8 T-cell frequencies peak at 5 days post-RT and then gradually decline to pre-RT levels (34). Five-days post-RT also reflects the highest effector to Treg ratio suggesting an ideal time point for checkpoint blockade. These data further support RT dosing with hypofractionation in a limited number of fractions as additional fractions may ablate recently infiltrated lymphocytes. The work by Frey et al. reinforce these findings (35). They showed that following 5 Gy × 2 fractions, CD8 T-cells peak at day 8 and decline significantly by day 9 while Treg have a bimodal peak on days 8 and 10 (35). Together these studies suggest that while the exact T-cell tumor infiltration kinetics may vary depending on the murine model and RT dose, close sequencing of checkpoint blockade following RT should be the goal to take advantage of the peak in tumor effector CD8 T-cells.

### Sequencing Depends on the Checkpoint Agent

Optimal RT and immunotherapy sequencing may also depend on the immuno-modulatory agent utilized. As articulated earlier, anti-PD-L1 appears to have the greatest synergy with RT when administered concurrently (30). In contrast, Young et al. have shown data in support of pre-treating with a TGF-β inhibitor in a mouse model of multiple different cancer types including colorectal cancer (36). TGF-β is a factor critical for Treg differentiation, and it is capable of impairing CD8 T-cell effector function. Using a small molecule inhibitor of TGF-β, the authors found an increase in intra-tumoral activated CD8 T-cells and fewer CD4 Treg. For the colorectal cancer experiments, mice were treated with 20 Gy × 1 fraction 7 days after the initiation of the anti-TGF-β therapy. They demonstrated improved survival in mice pre-treated with anti-TGF-β and RT compared to RT alone.

More recently, the same group directly compared the sequencing of two different immuno-modulatory agents relative to RT. In this pre-clinical study, they first evaluated whether administering a CTLA-4 antagonist 7 days prior, 1 day following or 5 days following RT (20 Gy × 1 fraction) changed outcomes. The best outcomes were observed when anti-CTLA-4 was delivered before RT. Interestingly, they showed that all mice that cleared the tumors were resistant to re-challenge with the same cell line at 100 days, suggesting the development of T-cell memory. The group then tested sequencing of anti-OX40. OX40, a secondary co-stimulatory molecule expressed by activated T-cells, was stimulated with the same schedule as anti-CTLA-4 and the highest percent survival was seen in the 1 day post-RT group (37). The authors concluded that the effect of sequencing is dependent on the mechanism of the immunotherapy being used. Given that anti-CTLA-4 may act on naïve T-cells and Treg (38) and anti-PD-1 acts on newly activated and exhausted T-cells (6, 32, 39), these differences in optimal timing are not surprising.

**Figure 1:**
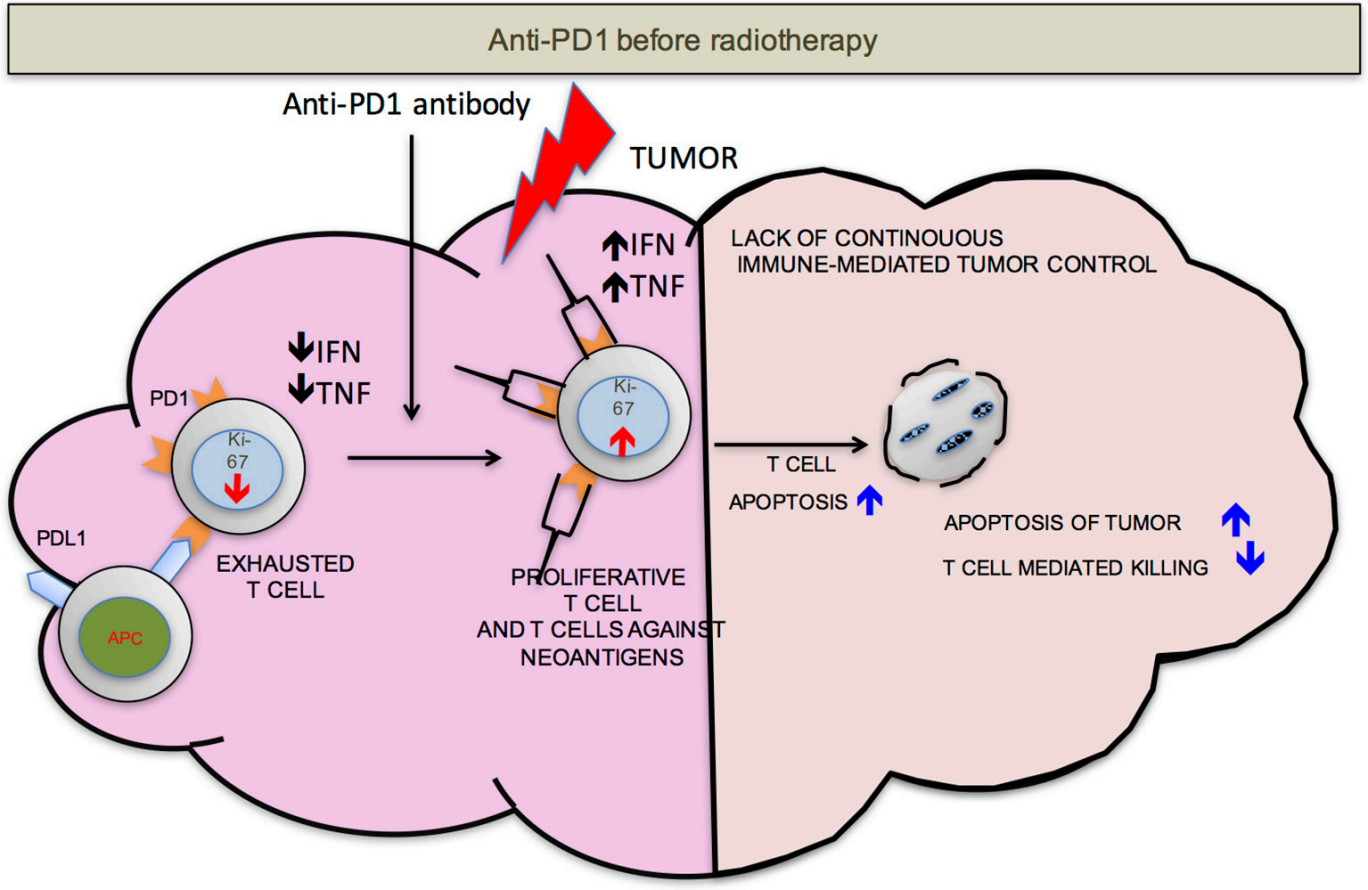
PD-1 before RT. Once the anti-PD-1 antibody is infused into the host, the exhausted cells start proliferating as detected by increase in Ki-67, become functionally active (increased production of IFN-γ and TNF-α) and traffic to the tumor from secondary lymphoid organs. Radiation delivered to the tumor at this point may be detrimental to the anti-tumor responses due to radiation induced T-cell apoptosis. This may compromise control.

**Figure 2:**
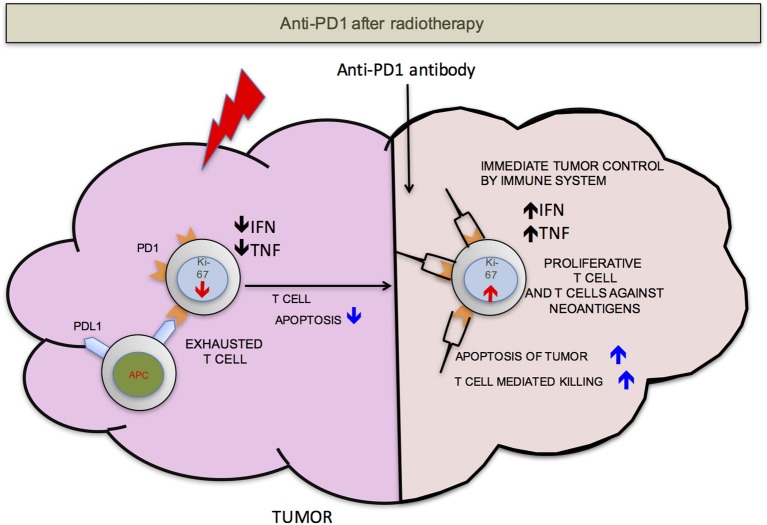
PD-1 after RT. If the radiation is delivered before anti-PD-1 therapy, there will be less T-cells apoptosis. T-cells that infiltrate after radiation mediated neo-antigens release have a better chance to mount an effective anti-tumor response. For simplicity, PD-L1 is shown on only antigen presenting cells (APCs).

We propose that ideally anti-PD-1/L1 and RT should be given concurrently but that if not RT should precede the administration of checkpoint blockade. RT delivered to the tumor following anti-PD-1/L1 may obliterate the recently infiltrated and reinvigorated T-cell response (Figure 1). In contrast, if RT is delivered before anti-PD-1/L1, RT stimulated naïve T-cell differentiation will synergize with checkpoint blockade and RT induced T-cell death of anti-PD-1/L1 reinvigorated T-cells may be avoided (Figure 2).

## RT Dose, Fractionation and the Immune Response

Varying dose and fractionation of RT in combination with immunotherapy and evaluating the anti-tumor immune response is an active area of investigation. Recent experiments from Morisada et al. in which primary tumor and abscopal tumor control rates were measured in a syngeneic mouse model of head and neck squamous cell carcinoma (SCC) following high-dose hypofractionated (8 Gy × 2) or low-dose daily fractionated (2 Gy × 10) RT in combination with concurrent anti-PD-1 showed that daily fractionated RT preserved peripheral and tumor-infiltrating CD8 T-cell accumulation and activation, reduced peripheral and tumor granulocytic myeloid derived suppressor cell (gMDSC) accumulation and did not impact Treg (40). Similarly, Type I IFN levels and expression of IFN-responsive MHC class I and PD-L1 was greater in those subjected to the daily low-dose fractionated regimen, and primary and abscopal tumor control improved when combined with anti-PD-1. Importantly, the local and abscopal effects appears to be similar for different hypofractionated regimens with similar biological equivalent dose (BED) (3 × 9.18 Gy in 3 or 5 days or 5 × 6.43 Gy in 10 days) (41).

Investigators have tested different total doses and fractionation schemes in a variety of pre-clinical models to maximize the abscopal effect (Table 1). Mice engrafted with the B16 melanoma cell line were treated with 15 Gy × 1 or 5 Gy × 3 fractions. The single fraction dose increased antigen availability and the number of tumor specific T-cells secreting IFN-γ in the tumor-draining lymph node to a larger extent than fractionated RT (42). They also showed that tumors receiving 15 Gy had greater infiltration of APCs and CD8 T-cells compared to 5 Gy × 3. To determine the dose for optimal tumor and immunologic response, Schaue et al. conducted a single fraction dose escalation study with doses from 5 to 15 Gy and demonstrated that doses of 7.5 Gy and above are immuno-stimulatory, defined by an increased number of tumor-reactive T-cells (43). However, at high dose, 15 Gy × 1, there was an increase in the splenic Treg fraction. They showed that if they instead fractionated the 15 Gy into 2–5 fractions, fewer Treg and more effector T-cells were identified in the spleens with an optimal dose fractionation of 7.5 Gy × 2. The authors do not offer a clear mechanism for the increased splenic Treg frequency at higher dose or whether this was mirrored in the tumor, but it may depend on the immunologic milieu generated by high dose fractions. Dewan et al. investigated dose and fractionation in a murine syngeneic breast cancer cell line subcutaneously injected at two distinct sites to assess the abscopal response. RT was delivered to one tumor site in 3 different regimens (20 Gy × 1, 8 Gy × 3, or 6 Gy × 5) with or without anti-CTLA-4 (44). The primary site and the secondary site were then monitored for response. They found that a significant abscopal effect was only induced when RT was administered with anti-CTLA-4 in either of the fractionated regimens. They concluded that a single dose, despite, or perhaps because of its size, was insufficient to induce an abscopal effect. These data taken together suggest that there is an optimal range (typically high dose per fraction) for the abscopal effect induction which is further supported by the new data concerning the cGAS-STING pathway.

**Table 1:** Studies on the effect of RT dose and fractionation on immune effect.

**Disease and animals**	**RT dose and fractionation**	**Findings**	**References**
Mice with OVA-expressing B16-F0 tumors	15 Gy × 1–3 fx	Single fx increased antigen availability and the number of T-cells secreting IFN- γ in the tumor draining LN to a larger extent that fractionated RT	(42)
Mice with B16-OVA	0–15 Gy × 1 fx; 15 Gy × 2, 3, or 5 fx	For single fx dose, tumor control increase with dose of RT. For 15 Gy, administration in 2 fx gave the best tumor control and tumor immunity.	(43)
Mice with TSA	20 Gy × 1, 8 Gy × 3, 6 Gy × 5	Abscopal effect occurred only in mice treated with the combination of immunotherapy and fractionated RT	(44)
Human SW480 colorectal tumor cells, *in vitro*	2 Gy × 5, 5 Gy × 3, 15 Gy × 1	Fractionated RT resulted in higher expression of IL-12p70, IL-8, IL-6, and TNF-α.	(45)

The importance of the cGAS-STING pathway on the anti-tumor immune response stimulated by both radiation and anti-PD1/L1 has now been established. cGAS (cGAMP synthase), a sensor of cytosolic DNA, a PAMP, catalyzes the formation of second messenger cGAMP which induces type I interferons via the adaptor protein STING. It was shown that cGAS –deficient mice bearing a B16 melanoma had a reduced response to anti-PD-L1 treatment relative to wild-type controls (46). In the cGAS knockout mice there was a decrease in the number of tumor specific CD4 and CD8 T-cells relative to wild-type anti-PD-L1 treated. The effect of anti-PD-L1 blockade was enhanced by intramuscular injections of cGAMP (cGAS product). In the RT context, it has been previously shown that type I interferons induced by RT are important for mediating the anti-tumor immune response (47). Deng at al. demonstrated that the STING signaling axis is activated in DCs, and cGAS is essential for the sensing by the DC of irradiated-tumor cell derived dsDNA. Additionally, they showed that STING promotes an anti-tumor CD8 T-cell response with an increased frequency of IFN-γ^+^ CD8 T-cells in the tumor-draining lymph node. Interestingly, there appears to be a link between radiation dose per fraction, the cGAS STING axis and radiation's synergy with immunotherapy. The exonuclease, TREX1, is upregulated by an RT dose per fraction greater than 10–12 Gy, and its expression degrades cytosolic dsDNA. This leads to a decreased synergy between radiation and immunotherapy (48, 49). This pathway is now a focus of ongoing and active investigation.

The upregulation of checkpoint molecules, the target of anti-PD-L1, can be induced in the tumor following RT, and the magnitude and kinetics of the induction may vary by dose and fractionation. 10 Gy in 5 fractions has been shown to robustly upregulate PD-L1 on CT26 tumors with a peak at Day 3 post-RT completion (30). In another elegant study, Derer et al. investigated the impact of RT, chemotherapy, and chemoRT on PD-L1 expression in a variety of murine tumor cell lines and found that standard fractionation and hypofractionated RT led to significant increases of PD-L1 expression in both melanoma and glioblastoma cell lines (50). *In vivo*, fractionated RT with dacarbazine induced PD-L1 expression on B16-F10 tumors, but not RT alone. In the context of human rectal cancer, Lim et al. evaluated pre chemoRT biopsies and post-chemoRT surgical specimens for expression of PD-L1 (51). The chemoRT regimen consisted of 50.4 Gy of radiation in 28 fractions with concurrent 5-fluorouracil and capecitabine. They found that PD-L1 is induced on tumor cells following chemoRT. Interestingly, if they then divided patients into 4 PD-L1 groups based on their biopsy and surgical expression levels, they showed that patients with high levels on biopsy and surgical specimens had the shortest overall survival. Importantly, however, patients that went from low to high levels of PD-L1 did not have shorter survival times suggesting that the PD-L1 induction by chemoRT is not deleterious and may provide an additional opportunity for checkpoint blockade.

On occasion, it is difficult or impractical to deliver this higher dose per fraction ideal for eliciting an anti-tumor immune response. Under these circumstances, the RT may be delivered by irradiating a fractional tumor volume, thereby reducing adverse effects. Using a 3-dimensional lattice radiation therapy (LRT) system, we have shown in a preclinical abscopal model that 20% volume irradiation (delivered to two 10% volumes) of the tumor resulted in significant growth delay in both the irradiated and unirradiated tumors (52). These abscopal effects were mediated by the down-modulation of T_H_2 functions and induction of robust IFN-γ and T_H_1 response in addition to increased T-cell infiltration and expression of TRAIL in the irradiated and unirradiated tumors (52). Interestingly, significant radiation-induced abscopal effects were observed in two of seven patients where only the hypoxic region of the tumor was irradiated with a single fraction of high dose radiation (53, 54). Immunomodulatory effects of the treatment were not assessed. These studies suggest that by partial irradiation of tumor volumes, high doses of radiation can be delivered with enhanced immunomodulatory potential, however, more studies are required to examine these novel approaches.

**Figure 3:**
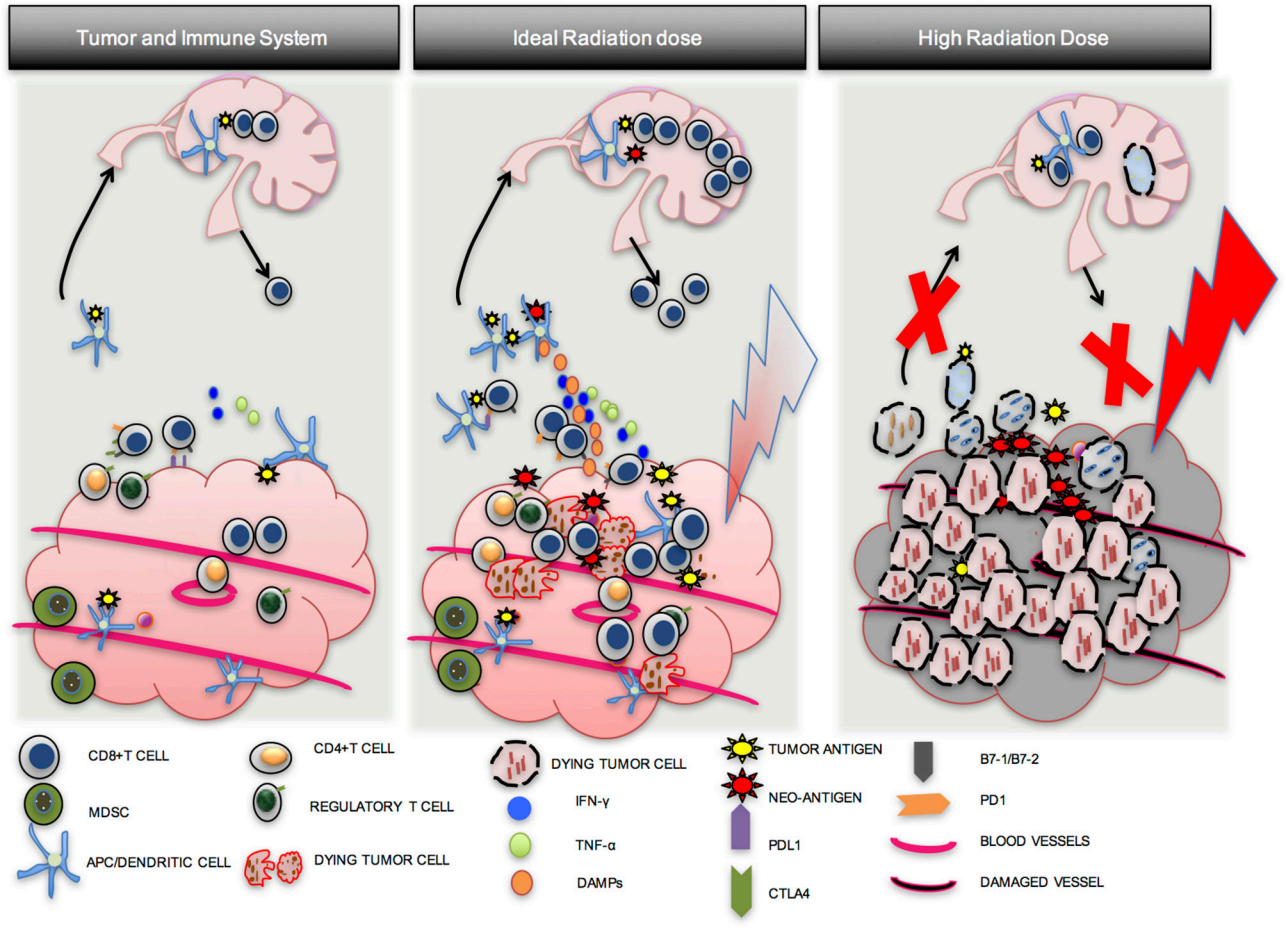
Importance of optimal radiation dose. A number of factors lead to the establishment of tumors in a host. Although CD8 T cells may be present in the tumor, they are exhausted, may not be able to find tumor cells that they were activated against, or there may be an immune suppressive environment induced by multiple cell types (1st Column). An ideal dose of radiation will induce inflammatory tumor cell death and activate an anti-tumor T-cell response via APC maturation (2nd Column). A high dose of radiation may induce tumor cell death but may also damage blood vessels and induce more CD8 T cell apoptosis. Local control from the direct effects of RT may be good, but effective immune priming and distant control may be compromised (3rd Column).

In summary, optimal radiation dose appears to be somewhere between 8 and 10 Gy per fraction in 1–3 fractions, and appears to be critical to an effective anti-tumor response. Although CD8 T-cells may be present in a tumor prior to RT, they may be downregulated by PD-L1-PD-1 mediated immune exhaustion, may not be able to find tumor cells that they were activated against, or there may be an immune suppressive environment induced by multiple cell types. An ideal radiation dose will induce tumor cell mitotic catastrophe (IDC), release tumor neo-antigens and endogenous adjuvants, increase APC maturation and antigen presentation, increase CD8 T-cell proliferation and migration to the tumor, and lead to effective anti-tumor response. A sub-optimal radiation dose may be effective in activating CD8 T-cells, but will fail to achieve standard of care treatment goals such as local control. An excessively high dose will induce tumor cell death and improve local control, but may also damage normal tissue and tumor vasculature with the added disadvantage of inducing widespread CD8 T-cell apoptosis, compromising immune priming, distant control, and the opportunity for induction of the abscopal effect (Figure 3) (55).

## Clinical Data

### Dose, Fractionation and Sequencing

The earliest and best trial evaluating different RT doses and immunotherapy was published in 2012. This phase I trial combined three different doses of stereotactic body radiotherapy (SBRT) and IL-2 where tumor and immune responses were evaluated in patients with metastatic melanoma or RCC. Patients received one of three regimens: SBRT 20 Gy × 1, 2, or 3 fractions on a Monday, Wednesday, and Friday schedule, followed by high dose IL-2 (600,000 IU) on the following Monday (72 h after completion of RT). The authors observed an objective response of 66% (8 of 12 patients with a complete or partial response) as measured in the Response Evaluation Criteria in Solid Tumors (RECIST). Additionally, the responding patients had a higher frequency of proliferating effector memory CD4 and CD8 T-cells without a difference in the frequency of proliferating Treg (56). They concluded that SBRT and IL-2 could be administered safely. Interestingly, they did not demonstrate a relationship between SBRT dose and overall response, however, the very small number of patients in this trial and the use of IL-2 allow limited conclusions to be drawn. Additionally, as described previously, the specific immuno-modulatory drug administered with RT is expected to influence optimal timing as well as dose. Finally, it is also well known that different tumor histologies have different radiosensitivities (57), therefore, it is conceivable that the optimal dose for antigen release and immunologic activation is tumor specific. Despite these limitations, this is a landmark trial with one patient with widely metastatic disease achieving PET complete response—an abscopal effect.

More recent studies have confirmed the safety of combination checkpoint blockade and RT without supporting a specific RT dose or RT/checkpoint sequencing (58, 59). An exciting study out of the University of Chicago showed an increased immune score (median expression level of normalized pre-selected genes) in the irradiated metastasis correlated with a greater change in the unirradiated lesion (60). The dose used varied from 30 Gy in 3 fractions to 50 Gy in 5 fractions determined by anatomic site with anti-PD-1 given every 3 weeks and initiated within 7 days after the final SBRT fraction. Additional data suggests synergy between RT and anti-CTLA-4 or anti-PD-1 in metastatic castration resistant prostate cancer and advanced non-small cell lung cancer (NSCLC), respectively (61, 62). The majority of on-going clinical trials prescribe concurrent administration of immunomodulatory agents and RT guided by the preclinical data (63). Of note, the recently published PACIFIC trial of stage III NSCLC demonstrated an overall survival benefit to adjuvant durvalumab (anti-PD-L1) following chemoradiation (64). As NSCLC has a very high rate of distant failure, this suggests that durvalumab improved local control as well as the micrometastatic disease (abscopal effect). There is now an actively enrolling trial evaluating the benefit of concurrent durvalumab with chemoradiation in stage III NSCLC (NCT03519971).

Data from the brain metastasis literature also supports close sequencing of RT and checkpoint blockade, although most of these data evaluate local control rather than an abscopal response. In one of the larger retrospective analyses, 75 melanoma patients with 566 brain metastases were evaluated. They received SRS and immune checkpoint therapy between 2007 and 2015 at Yale University (65). SRS was given in a single fraction to a median of 20 Gy (range, 12–24 Gy). Seventy-two percent of patients received anti-CTLA-4 and 28% received anti-PD-L1. Fifty-five percent of lesions were treated with concurrent SRS and immunotherapy (SRS administered within 4 weeks of immunotherapy). It was shown that, compared to non-concurrent treatment, concurrent use of immunotherapy and SRS resulted in a significant greater median percent reduction in lesion volume. Another study of 46 patients with metastatic melanoma who received ipilimumab and SRS found that patients treated with SRS during or before ipilimumab had higher overall survival and less regional recurrence suggesting an abscopal response compared to those treated with SRS after ipilimumab (66).

In totality, these data as consistent with preclinical results and our model (Figures 1, 2) that the concurrent use of RT and immunotherapy, results in a more pronounced treatment response.

Data supporting concurrent administration with RT for agents other than checkpoint inhibitors has also been evaluated. Forty-One patients with metastatic solid tumors treated with concurrent RT and granulocyte-macrophage colony-stimulating factor (GM-CSF) had stable or progressing metastatic solid tumors with at least three measurable metastatic sites and were on single chemotherapy or hormonal therapy (14, 67). Of the 41 patients, the most common tumor types were non-small cell lung cancer (44%) and breast cancer (34%). Two metastatic lesions were sequentially treated in each patient. Each lesion received 35 Gy of RT in ten fractions in two consecutive weeks, with daily subcutaneous GM-CSF injections lasting for 2 weeks starting during the second week of RT. The same process was repeated for the second metastatic lesion. Abscopal response (here defined as at least 30% decrease in the longest dimension of the best responding lesion) was observed in 19 (46%) patients. This is despite a non-optimal protracted regimen of 35 Gy in 10 fractions. Fourteen grade 3 or 4 toxicities attributable to either RT or immunotherapy were observed, with fatigue being the most common.

Finally, to date, at least 46 RT-induced abscopal effect case reports have been published from 1969 to 2014 (2). Table 2 displays a selection of representative studies. Histologies that have demonstrated abscopal effects include hepatocellular carcinoma, adenocarcinoma of the lung and esophagus, medullary thyroid carcinoma, Merkel cell carcinoma, follicular lymphoma, lymphocytic lymphoma, Hodgkin's lymphoma, CLL, renal cell carcinoma, and melanoma. Of the reported cases, the median age was 64 years (range: 28–83), the median RT dose was 31 Gy (range: 0.45–60.75), and the median dose per fraction was 3 Gy. The median time to an abscopal effect was 2 months (range: 0–24 months) and the median time to progression was 6 months (range: 0.7–14 months). Of these 46 published cases, only five patients had immunotherapy during treatment, four of which were melanoma patients. Therefore, relying on currently published case reports to guide timing of RT with immunotherapy is difficult. However, what can be gleaned from these case reports is that the abscopal effect does occur in multiple different cancer histologies.

**Table 2:** A selection of clinical trials and case reports that evaluated immune-stimulatory effects of RT.

**Disease and patients**	**RT doses**	**IO**	**Sequence**	**Immune effects**	**Toxicity**	**References**
12 patients with Stage IV melanoma or renal cell carcinoma	SBRT, 1–3 fx, 20 Gy/fx	IL-2	IO given 3 days after RT	8 (66%) patients achieved CR (*n* = 1) or PR (*n* = 7)	No DLTs attributable to SBRT	(56)
41 patients with metastatic solid tumors	35 Gy in 10 fx	GM-CSF	IO started during second week of RT	Abscopal effects occurred in 19 (46%) patients	13 Grade 3 and 1 grade 4 adverse events attributable to RT or IT	(14)
Patient with metastatic solid tumors	SBRT, 3–5 fx, 10–15 Gy per Fx	Pembro	IO given within 7 days after final SBRT	Correlation between immune score in irradiated tumor and size decrease in unirradiated tumor	3 Grade 3 pneumonitis, 2 Grade 3 colitis, 1 Grade 3 hepatitis	(60)
HCC patient treated to a large cranial lesion	30 Gy	None	N/A	Abscopal effect was observed in primary and un-irradiated bone lesions after 10 months	Not reported	(68)
NSCLC patient with bone and adrenal metastases	2 Gy × 30 fx and 26 Gy × 1 fx to 2 different lung lesions	None	N/A	Abscopal effect was noted in bone and adrenal metastases after 12 months	Not reported	(69)
Follicular lymphoma patient	36 Gy in 26 days to paraaortic and pelvic lymph nodes	None	N/A	Abscopal effect was observed in liver, spleen, axillary lymph nodes	Not reported	(70)
Patient with metastatic RCC	20 Gy in 10 fx to the right kidney	None	N/A	Abscopal effect was observed in paratracheal nodes and bilateral pulmonary nodules	Not reported	(71)
Patient with metastatic melanoma	28.5 Gy in 3 fx to paraspinal mass	None	N/A	Abscopal effect occurred in the right hilar and splenic lesions	Not reported	(3)

*RT, radiotherapy; SBRT, stereotactic body radiotherapy; IO, immunotherapy; HCC, hepatocellular carcinoma; RCC, renal cell carcinoma*.

## Discussion

Many of the topics addressed in this review remain areas of active inquiry with a number of smaller checkpoint and RT studies having been published. Our lab is also investigating questions of fractionation and timing. Although there appears to be a consensus that hypo-fractionation is superior to conventional fractionation, the optimal dose for an abscopal or local immune response may depend on tumor histology and non-synonymous mutation burden due to varying radio-sensitivities and neo-antigen load (72). Additionally, the optimal interaction may also vary with the specific immunotherapy administered as CTLA-4 and PD-1/PD-L1 antagonists have distinct and non-redundant mechanisms. These numerous variables add complexity to any proposed clinical trial design.

We recommend including different fractionation schemes in any proposed immunotherapy and radiation clinical trials and suggest potentially varying the fractionation schemes from one tumor histology to another. These data suggest that a dose per fraction of close to 10 Gy with 1–3 fractions is likely optimal for abscopal effect induction. Importantly, a dose and fractionation regimen optimized for a robust local response may be expected to differ from that optimized for a distant abscopal response and additional data are needed to elucidate these likely tumor-specific thresholds.

We also encourage further investigation involving the sequencing of radiation and immunotherapy. Evidence presented here suggests immunotherapy should be initiated at the start of radiation when employing single or high dose per fraction RT as this is the time when a bolus of neo-antigens is released, followed later by more limited T-cell epitope availability. Conventional fractionation may instead lead to a steady release of tumor antigens throughout treatment and the exact point of immunotherapy initiation may be less critical, although earlier initiation of immunotherapy is likely to remain superior. Finally, the mechanism of radiation and immunotherapy for T-cell activation is specific (29), and understanding why close sequencing rather than more remote administration of immunotherapy improves control in several contexts is an important avenue of investigation.

## Conclusion

The synergy between RT and immunotherapy has now definitively entered the mainstream. A deep and clear mechanistic understanding of RT's immune system stimulation and its synergy with immunotherapy affirms the value in pursuing and expanding this avenue of research. There are, however, still many unanswered questions in the optimization of the abscopal response including, but not limited to: RT and immunotherapy sequencing, RT dose and fractionation, and RTs specific interactions with different immuno-modulatory agents and individual tumor subtypes. It is our hope that the research community continues to vigorously pursue these and other vital questions surrounding the induction of the abscopal effect. The solution to transforming RT from a purely local or palliative therapy to a treatment important for long-term metastatic control, we believe, may lie in the answer to these questions.

## Author Contributions

ZB, JW, SG, and SZ: wrote the manuscript; TN: created the figures; SZ: created the tables; WM, MK, and SK: edited the manuscript and provided expertise.

### Conflict of Interest Statement

The authors declare that the research was conducted in the absence of any commercial or financial relationships that could be construed as a potential conflict of interest.
